# Signs of Anxiety and Salivary Copeptin Levels in Dogs Diagnosed with Separation-Related Problems in a Short Separation Test

**DOI:** 10.3390/ani12151974

**Published:** 2022-08-03

**Authors:** Ludovica Pierantoni, Mariangela Albertini, Patrizia Piotti, Giulia Ripamonti, Paola Pocar, Vitaliano Borromeo, Federica Pirrone

**Affiliations:** 1 Veterinary Behaviour & Consulting Services at CAN Training Centre, 80128 Naples, Italy; ludovica.pierantoni@gmail.com; 2 Department of Veterinary Medicine and Animal Sciences, University of Milan, Via dell’Università, 6, 26900 Lodi, Italy; mariangela.albertini@unimi.it (M.A.); giulia.ripamonti27@gmail.com (G.R.); paola.pocar@unimi.it (P.P.); vitaliano.borromeo@unimi.it (V.B.); federica.pirrone@unimi.it (F.P.)

**Keywords:** dogs/canine, stress, separation-related problems, copeptin, anxiety

## Abstract

**Simple Summary:**

Up to 56% of the general dog population show signs of separation-related problems, a group of problems characterized by highly variable phenotypes on which research, to date, has mostly provided contradictory findings. In the present study, we analyzed the behavior and salivary concentrations of copeptin, a biologically stable biomarker of stress, in dogs with separation distress and in dogs with no problems. The animals were tested before, during and after three-minute separation from the owner, in a new environment. Dogs in the two groups differed significantly in their activity levels and stress-relief activities during the three observation phases. In addition, a different tendency in the two groups was found in salivary copeptin concentration behavior.

**Abstract:**

The need for faster diagnosis and more accurate treatment decisions in separation-related problems (SRPs) in dogs is urgent, and a more precise behavioral phenotyping and the development of biomarkers may be of great value. Vasopressin could be a potential non-invasive biomarker of anxiety in dogs with SRPs, but reliable measurement of its concentration is challenging. Here, we compared the behavior and salivary concentrations of copeptin, an arginine vasopressin surrogate, in dogs with SRPs (Case group, n = 13) and with no problems (Control group, n = 15) as they were introduced to a novel environment and subjected to a short episode of separation and reunion with the owner. Dogs in the Case group had greater odds of showing locomotory or oral behaviors during the pre- and post-separation than Controls, while the odds were significantly lower during separation. They also had greater odds of being persistent in seeking attention and proximity from the stranger during reunion. Overall, dogs with SRPs were more likely to express an anxiety-like state during the entire test than Controls, with separation from the owner, and even its anticipation, possibly accounting for this group difference. Although salivary copeptin concentrations did not differ between the two groups, a different trend was detected in Cases and Controls that is worth exploring in further validation studies involving a larger sample.

## 1. Introduction

More and more people all over the world live with a dog with whom they establish an indissoluble emotional and affective connection, to the point of considering their dog a family member within the household [1]. However, the human–dog relationship also has a dark side, that is represented by the abandonment or relinquishment of the animal. A major cause of this relationship’s failure is dog behavior problems, such as separation-related problems (SRPs) [2]. SRPs are among the most common behavior disorders in family dogs [3], and the anxiety emotional system is recognized as one possible cause. According to Karagiannis et al. [4], up to 56% of the overall dog population may suffer from clinical symptoms of SRPs at some point in their life, which represents approximately 85 million dogs with SRPs in the US and Europe taken together. Although a dog can be expected to exhibit mild distress in the absence of, or lack of access to, the owner, as a function of the dog–owner attachment bond [5], in some cases, levels of stress are so intense that they pose serious challenges for both the dogs’ and the owners’ welfare [6,7]. The stress-related signs can be highly variable [8], and may be physical, physiological and behavioral. Both specific and non-specific behaviors have been observed, including destruction of objects, escape attempts, intensive vocalization and salivation or inappropriate urination and defecation [8]. The authors are aware that the individual variability with which these signals can be manifested [8,9], in terms of strength, dynamics and timings, still leaves many questions open. For example, some affected dogs are reported to destruct during owner absence, while other do not show destruction [10]. It has been hypothesized that multiple underlying motivations and emotions, including anxiety, are behind SRPs [11,12]. SRPs are often underestimated and underdiagnosed due to misinterpretation of normal and “pathological” anxiety [13], the latter defined here as the excessive or maladaptive response of a subject to potential threats that may impair their homeostasis [14,15], and the scarce awareness of mild clinical signs by owners [16]. Early recognition of pets suffering from SRPs could benefit from identification of specific measurable biomarkers for stress and/or anxiety in dogs. Unfortunately, the physiological correlates of the various phenotypes of SRPs are still poorly described in scientific literature. The neuropeptide vasopressin (AVP) has been shown to augment anxiety and fear expression [17] and to increase the neuroendocrine stress response [18] following social separation in both rodents and humans [17,19,20]. In dogs, saliva concentrations of AVP had been studied in relation to fear and aggression [21,22]. A previous study has revealed the potential role of AVP in anxiety-like behavior in dogs [23]. In particular, an increased salivary AVP level was detected immediately after separation-induced social stress in dogs diagnosed with anxiety-related SRPs compared to unaffected dogs. However, obtaining a reliable measurement of AVP concentration can be very challenging and prone to analytical and pre-analytical errors [24]. More recently, great attention has been devoted to copeptin (CoP), a 39-amino acid glycopeptide that comprises the C-terminal part of the AVP precursor (CT-proAVP) that was found to be a stable and sensitive surrogate marker for AVP release [25]. Unlike vasopressin, copeptin is stable for several days after blood withdrawal [26]; it is found in higher concentrations in blood because it is unbound to blood platelets [24], its detection does not require extraction processes or other complex pre-analytical steps [27] and it can be easily measured with a chemiluminescence test using a minimal volume of biological sample [28].

In this study, we examined the behavior of dogs either diagnosed with anxiety-related SRPs (Case group) or without behavior problems (Control group) during a test based on a short separation from the owner that had previously been employed to investigate distress biomarkers [23]. This behavioral test simulates a commonly occurring human–dog separation, that might potentially induce anxiety in affected dogs. We aimed to answer the following question: is there a different behavioral reaction in dogs before, during or after separation depending on the group (Case vs. Control)? From previous preliminary findings [23], we expected that dogs with a previous diagnosis of SRPs would show more anxiety-related behaviors when separated from the owner in an unfamiliar environment. Here, we have gone a step further, providing preliminary observation of salivary copeptin fluctuations with the intent to understand if, in case the difference in behavior is confirmed, the neuropeptide might moderate this relationship.

## 2. Materials and Methods

### 2.1. Participants and Study Setting

This study was part of a research project designed to investigate distress and anxiety in pet dogs brought by their owner to the Veterinary Behavior and Consulting Services at Comportamento Animale Napoli (CAN) Training Center in Naples, Italy, by checking for signs of fear or anxiety-related conditions. The target sample size for the study was determined based on a power analysis with 80% power, a large effect size (0.50) and an α value (error rate) of 0.05. Details on participants’ recruitment and study protocol have been previously described [23]. Briefly, 13 dogs were selected by convenience sampling from those who had received a diagnosis of an anxiety-related SRP based on the outcome of the behavioral consult by a Diplomate of the European College of Animal Welfare and Behavioural Medicine (Case group). We included dogs that showed signs of anxiety as soon as there was a real or virtual separation from the owner (Table S1). A diagnosis of separation-related problems was made when a dog showed consistent destruction, elimination, vocalization or salivation exclusively during the virtual or actual absence of the client. In addition, a videotape of the dog while alone was requested and analyzed, therefore it was also possible to make a diagnosis for those dogs that exhibited signs such as immobility, trembling, panting or pacing while in their owner’s absence, according to Overall, 2013 [6]. N = 15 Controls were selected from dogs whose owners attended the training center for the first time. The selected Controls were rated as behaviorally typical based on the outcome of a consultation purposely performed to include them in the present study. The two groups were similar with respect to the dogs’ age, sex, sexual status and breed type. The dogs in the Case group had an average age of 47 months (9.9 SE, min–max 12-108), and included 6 females (1 spayed) and 8 males (5 neutered) and 6 were of pure breed. The dogs in the Control group had an average age of 37 months (SE 6.9, min–max 12-108) and included 6 females (3 spayed) and 9 males (4 neutered), and 8 were of pure breed. All dogs attended the training center for the first time.

Moreover, inclusion criteria required that Cases and Controls had belonged to their current owners for at least 8 months to ensure that they had a reasonable amount of time to form a relationship with the owners and were an adult at the time of the study. All dogs underwent a physical examination as well. The Case group also had blood tests (a complete blood count and a serum chemistry profile) within 10 days after the test to exclude any medical condition that could justify the symptoms. Those who were healthy and not yet in treatment for behavioral or physical problems at the time of data collection were included in this study. In particular, the behavioral visit allowed us to exclude dogs with a history or present signs of any SRP comorbidities, especially those that could have dramatically impacted on the test, such as stranger-directed aggression or fear of strangers or novel environments. Exclusion criteria included estrus, pregnancy and nursing. 

As previously described [23], testing took place during the behavioral consult in a 300 m^2^ outdoor arena that had four sides and was enclosed with chain-link fencing approximately 2 m tall; one side of the arena included an entrance gate. The arena contained three chairs (for the owner, the veterinary behaviorist and a cameraperson), a bowl for fresh water and some toys(Figure S1). All objects were thoroughly washed after each test to eliminate any olfactory interference, similar to the procedure of Scandurra et al. [29].

The duties of the veterinary behaviorist and the cameraperson were always performed by the same two women, who had never met the dogs before. All dogs were tested in mild weather conditions and in the late afternoon (between 16:30 and 18:30), after the center’s planned closure, so no other social stimuli were present. The same procedure was used for dogs in both groups.

The protocol involved 3 different phases: (1) a 10 min acclimatization phase, prior to the short separation task, during which the owner, the dog, the veterinary behaviorist and the camerawoman were together in the arena. The dog was left unleashed and free to explore the environment, while people remained seated in the chairs. Saliva was collected from the dog at the end of the tenth minute (T0); (2) a 3 min separation phase, during which the owner left the arena, whereas the dog remained in the company of the two strangers. The veterinary behaviorist responded in a friendly and reassuring way to any requests for interaction from the dog, including gently petting him/her and speaking to him/her in a calm tone. However, the dog could lead these interactions and was always free to disengage and move away from her. If a dog exhibited signs of severe distress or anxiety, the owner was asked to come back, and testing was stopped. This happened once and the dog was excluded from the study participants. At the end of the third minute, the owner returned to the arena, and the second sample of saliva was collected (T1); (3) a 10 min reunion phase, at the end of which saliva was collected (T2), and the test ended. Upon reunion, the owner was allowed to respond to their dog’s greeting by interacting both verbally and physically in a calm way.

### 2.2. Behavioral Observations

Observer-blind analysis of behavior was carried out with focal animal sampling and continuous recording using the behavioral coding software Solomon Coder [30]. Another coder, expert in animal behavior and unfamiliar with the aims and conditions of the study, independently coded 20% of the videos for reliability. A Cronbach’s α of 0.80 or higher was deemed acceptable for this study. Both social and individual behaviors were analyzed (Table 1). Behavior definitions were formulated based on a literature review [23]. For each behavioral variable, we measured the relative frequency (the number of occurrences per minute) and/or duration (time spent on a behavior, expressed in seconds) of occurrence during each observation period. Despite attempts to keep each phase duration consistent, the time could vary for a given phase. Thus, the behavioral duration data were converted to duration percentage, to allow comparison between phase sets and across data collection sessions.

### 2.3. Endocrine Measurements

Saliva samples were collected from dogs by the veterinary behaviorist with commercially available swabs (SalivaBio Children’s Swab, Salimetrics, Carlsbad, CA, USA) as previously described (Pirrone et al., 2019). The collected saliva was refrigerated at 4 °C and then stored at −20 °C immediately after it arrived at the laboratory. At the time of analysis, the samples were thawed at room temperature and centrifuged at 3500 rpm for 15 min, according to the protocol for salivary samples. All samples were analyzed by a laboratory technician who was blinded to the hypotheses and conditions, using a commercially available enzyme-linked immunosorbent assay kit from BlueGene Biotech (Shanghai, China) designed for quantitative determination of copeptin in dogs. Samples were used as neat (undiluted) according to pre-experiment results and assay manufacturer recommendations. Each sample was prepared in duplicate, and concentrations were calculated using a Micro Read 1000, Global Diagnostic (Geel, Belgium) microplate reader according to the relevant standard curves (range 50 to 1000 pg/mL). The mean recovery was 98.5% ± 6.3. The average intra- and inter-assay coefficients of variation, respectively, were 4.5–5.9% and 6.8–8.2%. The assay sensitivity was 1 pg/mL. No significant cross-reactivity or interference with CoP analogues was reported. Three participants from the Case group were excluded from CoP analysis due to an insufficient amount of saliva in the samples.

### 2.4. Statistical Analysis

After testing continuous variables for normal distribution using Shapiro–Wilk, in case data were not normally distributed, transformations were performed by applying decimal logarithms (base 10). In the case of behavioral data, the assumptions for parametric testing were not met, therefore generalized linear models (GzLMs) with either Poisson loglinear or gamma with log link distribution were run for counts and scale responses as dependent variables, respectively. Each behavior was entered into a separate model as a dependent variable, while group (Case vs. Control) and timepoint (T0, T1, T2) were the independent variables. Salivary copeptin concentrations were included in the models as a covariate. A factorial design was chosen, which contained all factor and covariate main effects, as well as timepoint-by-group and group-by-copeptin interactions. The strength of the associations was expressed as odds ratio (OR) and 95% confidence interval (95% CI); *p*-values < 0.05 were considered significant. Finally, group differences in salivary concentrations of copeptin across the three timepoints were analyzed using a general linear model (GLM) with repeated measures. Statistical analyses were performed with SPSS 27.0 (IBM SPSS Statistics for MAC, Armonk, NY, USA).

## 3. Results

### Behavioral Responses

Inter-observer reliability was confirmed for all variables, with a Cronbach’s α of 0.999. All behaviors’ median percentage duration and relative frequency across the three timepoints are reported in Table 2 and Table 3. Mounting and pacing were not sufficiently expressed to be analyzed.

**Figure 1:**
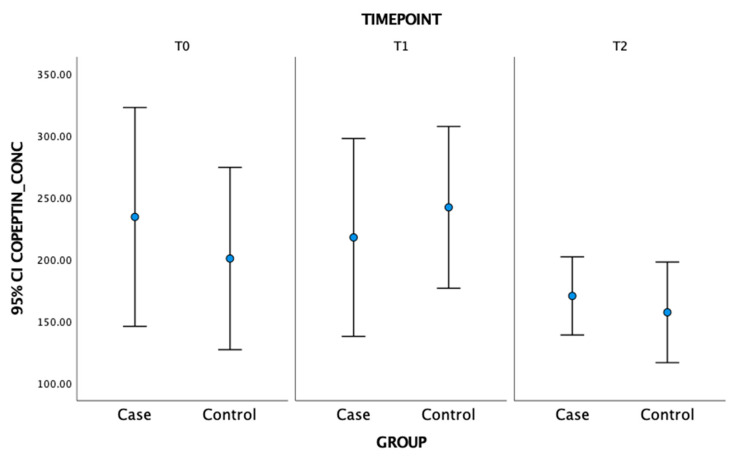
Mean concentrations of copeptin (pg/mL) measured in saliva before (T0), immediately (T1) and 10 min after (T2) separation from the owner in dogs from both groups (N = 25). No group differences across the timepoints were detected (*p* > 0.05).

Salivary copeptin concentrations are reported in Figure 1. No significant differences were found across the three timepoints between the two groups. GzLM model results are summarized in Table 4 and Table 5, where only statistically significant factors are reported. As for behaviors’ duration, the single significant predictor for explore and fence-directed behaviors was the timepoint. The three significant predictors for individual play were timepoint, group and the timepoint–group interaction, while the three significant predictors for spontaneous interactions with a stranger were timepoint, group and the group–copeptin interaction. The two significant predictors for jumps on owner were group and the group–copeptin interaction. The group–copeptin interaction always showed no or only a very weak effect on behavior, with the likelihood of showing the behavioral outcome being lower than 2%. As for behaviors’ frequency, the timepoint and the interaction between the timepoint and the group were significant predictors for exploring, fence-directed behaviors and restlessness. Timepoint, group, timepoint–group interaction and the interaction between the group and the concentrations of copeptin were significant predictors for individual play, Group and group–copeptin interaction were predictive of spontaneous interactions with a stranger and with the owner. Timepoint and group–copeptin interaction were significant predictors for drinking while whining was significantly predicted by timepoint, timepoint–group interaction and group–copeptin interaction.

## 4. Discussion

This study aimed to determine whether there were differences in specific behaviors between dogs which showed at-home separation-related problems caused by anxiety and dogs without behavior problems, while experiencing a potentially anxiogenic situation (brief separation from the owner in a novel environment and in the presence of two strangers). Moreover, we aimed to preliminarily explore whether the relationship between the diagnosis and the behavior of the dogs during the separation might have differed based on the salivary concentrations of copeptin, a 39-amino acid-long glycosylated peptide that is believed to mirror peripheral AVP levels.

We found a significant interaction between the group and the phase of the test (acclimatization, separation from owner, reunion) for most of the measured behavioral outcomes. The dogs in the Case group (affected by SRPs) were significantly more likely to exhibit individual play during both the acclimatization and the reunion phase compared to dogs in the Control group. In the present paper, individual play was defined as any motor behavior performed vigorously and directed towards an object, including chewing, biting, shaking from side to side and tossing objects using the mouth. Based on this definition, the authors are inclined to reckon that these could considered as stress-release activities. Many dogs are in fact reported to become hyper-active and display increased levels of locomotory or oral behaviors in response to acute stress [31]. This higher level of activity may be related to higher arousal in dogs affected by anxiety-related issues when dealing with novelties [6]. Supporting this possibility, during the pre- and post-separation phases, our Case dogs were also significantly more likely to engage in more frequent, but short, episodes of active investigation of the environment as compared to Controls, which is compatible with a state of anxiety [10]. Exploration and play are reported as being functionally similar, and possibly linked behavioral strategies for dealing with novelty, i.e., forms of coping mechanisms [32,33]. Conversely, the odds of individually playing among Case dogs were 64% lower than those among Controls during the separation phase. The lower odds of individually playing among Case dogs could thus be seen as a sign of a dog being behaviorally inhibited while their owner was absent. Then, when the owner returned, the SRP dogs could again use their coping strategies as well as release the tension provoked by anxiety. Consequently, their odds of displaying exploration and individual play increased again. In fact, they performed these behaviors even more than prior to separation, and there was an interaction between being in the Case group and the outcome “individual play” during this reunion phase (individual play frequency: OR = 2.81 vs. 4.92, acclimatization phase vs. reunion phase; individual play duration: OR = 5.12 vs. 5.69, acclimatization phase vs. reunion phase), which further support the possibility of the behavior being a coping strategy, assuming that the SRP dogs had accumulated more tension during the separation. This possibility is further supported by the fact that SRP dogs had significantly greater odds of whining and drinking, compared to Controls, as a possible indication of their level of distress in this phase. However, the dog–human bond may also play a role in the generation of this dynamic.

Another aspect that is worth commenting is that Case dogs were four times more likely than the Controls to be persistent in seeking attention and proximity from the stranger, namely the veterinary behaviorist, during the reunion phase. This behavioral pattern is consistent with that observed in a previous study with the same test [23] and could be seen as indirect evidence that the dogs in the Case group experienced a higher level of anxiety during the separation. During the separation phase, the veterinary behaviorist remained in the arena, and made attempts to reassure the dogs if they sought for attention or comfort. Case dogs were behaviorally inhibited during that phase, and her presence and efforts might have made the researcher a target of attention and exploration for them in the reunion phase, when the reduced anxiety could have led to reduced inhibition as well and to enhanced social interest. This result did not conform with what was found in the study by Topál et al. [5] in which dogs involved in a modified version of MDS Ainsworth’s Strange Situation Test (SST) [34] showed a tendency to seek and maintain contact with the returning owner but not the stranger, possibly expressing their willingness to spend more time in proximity to the owner. However, unlike our study, the presence of SRPs in the dogs of the study by Topal et al. [5] was not investigated, and therefore they might have been less prone to anxiety than our Case dogs. In addition, in the SST there are no reunion phases where the owner and the stranger are together with the dog, therefore even those dogs who had experienced distress during the separation from the owner might have never regained enough comfort to be able to engage in stress-releasing behavior.

As for salivary CoP concentrations, no significant group differences were detected. Copeptin concentrations were found to influence the relationship between the variable “group” (Case vs. Control) and some of the measured behaviors, regardless of the timepoint, but this interaction effect was too weak to be considered clinically relevant. Possible explanations for this result could be individual variability in the perception of the absence of the owner as a potential trigger for anxiety in the dogs or in the fluctuation of CoP concentration in different dogs in phases 1 and 2 of the test (as suggested by the wide variance in the CoP concentration in both groups), and/or actual absence of interaction of SRPs with copeptin variations as a contributor to anxiety. Specifically, the concentrations of CoP that we reported here did not reach statistical significance between the two groups overall; however, a different trend could be observed in Case and Control dogs which is worth commenting on: a trend towards a reduction was observed in both groups at T2, compared to T0, but at T1 copeptin concentrations increased in the Control group, while they were slightly decreased in the Case group. Copeptin has been shown to subtly mirror stress, potentially mediated via corticotrophin-releasing hormone. Specifically, in healthy human controls [35], corticotrophin-releasing hormone can stimulate copeptin release, that in turn shows a moderate correlation with ACTH concentrations. Demiralay et al. [36] also detected simultaneous release of copeptin and ACTH during acute stress. This phenomenon could be, therefore, at least partially responsible for an increase in copeptin concentrations observed in our study among Controls at T1, as saliva was collected immediately after the end of the separation period. No studies have been published about salivary CoP concentrations, despite the ever-increasing literature regarding this biomarker’s levels in serum. However, CoP is cleaved from the neurohypophysial hormone prepro-vasopressin and secreted in the blood during procession to vasopressin, whose time to reach saliva is quite well understood, appearing faster than other salivary hormones (e.g., cortisol) [22]. Previous studies have shown effects at a minimum time delay of 10 min [22,37] while other studies suggested that increases in salivary concentrations may be even earlier (e.g., 3 min) [38]. Given that stressors induce a rise in copeptin levels which, although non-specific, is proportional to their magnitude [39], it is possible that, in our study, the non-significant rise observed in salivary CoP at T1 reflected a successful coping response in Control dogs elicited at the start of the experiment in a novel environment. 

Case dogs showed decreasing concentrations of CoP across the test phases. Interestingly, studies in humans have shown a similar negative correlation between behavioral problems and physiological responses to stressful situations. For example, Hubert and de Jong-Meyer [40] found a decrease in salivary cortisol in response to a stressor, that was higher in very anxious subjects than in non-anxious controls. Two other studies revealed lower concentrations of plasma biomarkers, including ACTH and cortisol, under psychosocial stressful situations in anxious versus non-anxious people [41,42]. This, therefore, could suggest the existence of a negative relationship between neuroendocrine biomarkers and behavioral problems related to anxiety, as if the stress coping system was not sufficiently efficient in the anxious dogs. The neurobiological motivations have recently been demonstrated in humans confronted with chronic stress [43]. Under this condition, the bed nucleus of the stria terminalis would play a role in anxiety processing, becoming stress-inhibitory rather than stress-excitatory through inhibition of paraventricular nucleus (PVN) vasopressin-releasing neurons.

This study has some limitations, including the small sample size and the impossibility to collect blood analyses from healthy Controls, due to ethical restrictions. Moreover, as mentioned in the Introduction, the testing was interrupted for dogs showing excessive signs of distress and they were excluded from the study. Therefore, there might be no exploration of individuals with very high stress severity during separation from the owner. Although this happened only once in our case, the findings may not be generalizable directly to individuals with severe separation-related problems.

## 5. Conclusions

In conclusion, due to the ethological and physiological complexity of SRPs, studies aimed to determine the relationship between canine behavioral responses and peripheral concentrations of predictive biomarkers are extremely necessary and urgent. This study showed that a short separation from the owner, followed by the reunion, in a novel environment elicits peculiar behavioral responses in dogs who suffer from separation-related problems indicative of anxiety at home. Specifically, dogs with anxiety-related SRPs are prone to exhibit anxiety-like responses to separation, and to anticipation of it, which could be underestimated and need to be distinguished from SRPs caused by boredom (play), frustration, fear of noises, etc. [8,11,12]. Moreover, in SRP dogs, a trend was also seen towards a reduction in CoP salivary levels. This is a preliminary result, which, if confirmed, could suggest an anxiety-associated dysregulation of HPA-axis activity, mediated through vasopressin-dependent mechanisms. 

## Figures and Tables

**Table 1:** List of behaviors and definitions used in the study. F = frequency (number of occurrences); D = duration (s).

Behaviors	Description	Measured Values (F/D)
*Social behaviors*		
Jumping up	Both dog’s forelegs are out of contact with the ground, regardless of the position of the hind legs; the dog is in proximity to a person. The dog might also be entirely on the lap of a person (the owner or the stranger)	F, D
Spontaneous interactions	Staying close to and seeking attention and physical contact (nuzzling or pawing for attention, soliciting petting) from the owner or the stranger	F, D
Mounting	Sexual mounting of people or inanimate objects	F, D
*Non-social behaviors*		
Explore	Activity directed towards physical aspects of the environment, including sniffing, visual inspection and gentle licking	F, D
Individual play	Any behavior performed vigorously or at a galloping gait and directed towards an object when clearly not interacting with any human; these play behaviors include chewing, biting, shaking from side to side and tossing objects using the mouth	F, D
Fence-directed behaviors	Standing by the fence: standing close to the fence (<1 m), regardless of whether the face is oriented towards the exit; attention oriented towards the fence: staring fixedly at the fence, either when close to it or from a distance; behaviors oriented towards the fence: all activities resulting in physical contact with the fence, including scratching the gate with the paws, jumping on the fence and pulling on the fence with the forelegs or mouth	F, D
Restlessness	Changes in body position and state of locomotion indicating that the animal is uncomfortable	F
Drinking	Taking in fluids by lapping up water from the bowl with the tongue	F
Whining	High-pitched vocalization	F
Pacing	Increased motor activity, walking or running around without exploring the environment	F

**Table 2:** Median duration (in percentage, minimum–maximum) of behaviors expressed by dogs across the three timepoints.

Group	Timepoint	Jumps on the Owner	Jumps on the Stranger	Spontaneous Interactions with the Owner	Spontaneous Interactions with the Stranger
Case	A	0.00 (0.00–22.11)	0.00 (0.00–1.05)	7.083 (0.00–52.39)	4.88 (0.00–29.34)
Control	A	0.00 (0.00–2.05)	0.00 (0.00–11.25)	2.734 (0.00–98.63)	2.95 (0.00–7.90)
Case	S	n/d	0.31 (0.00–10.34)	n/d	8.97 (0.00–38.20)
Control	S	n/d	0.000 (0.00–23.09)	n/d	2.82 (0.00–68.06)
Case	R	0.87 (0.00-4.98)	0.10 (0.00–6.34)	9.97 (0.00–66.44)	8.74 (0.00–53.37)
Control	R	0.22 (0.00-2.77)	0.00 (0.00–15.65)	7.81 (0.21–42.40)	2.38 (0.00–13.63)
**Group**	**Timepoint**	**Explore**	**Individual play**	**Fence-directed behaviors**	
Case	A	63.12 (29.42–82.35)	1.461(0.00–29.50)	32.12 (2.17–171.06)	
Control	A	63.68 (45.29–96.58)	0.95 (0.00–33.10)	29.01 (0.00–171.06)	
Case	S	9.82 (0.00–31.23)	0.00 (0.00–2.79)	18.46 (1.53–156.24)	
Control	S	12.88 (0.00–53.10)	0.00 (0.00–16.47)	43.79 (0.06–138.74)	
Case	R	45.52 (7.36–69.13)	2.04 (0.00–28.58)	76.90 (2.21–167.67)	
Control	R	57.94 (22.63–79.42)	0.00 (0.00–55.71)	33.85 (0.27–137.56)	

A: acclimatization phase, S: separation phase, R: reunion phase.

**Table 3:** Median (minimum–maximum) relative frequency of behaviors expressed by dogs across the three timepoints.

Group	Timepoint	Jumps on the Owner	Jumps on the Stranger	Spontaneous Interactions with the Owner	Spontaneous Interactions with the Stranger	Explore
Case	A	0.00 (0.00–0.30)	0.00 (0.00–0.29)	0.45 (0.00–0.14)	0.40 (0.00–0.88)	1.57 (0.69–3.30)
Control	A	0.00 (0.00–0.30)	0.00 (0.00–0.79)	0.23 (0.00–0.89)	0.32 (0.00–0.98)	1.87 (0.70–4.15)
Case	S	n/d	0.12 (0.00–3.00)	n/d	1.31 (0.00–3.81)	1.41 (0.00–2.77)
Control	S	n/d	0.00 (0.00–2.71)	n/d	0.577 (0.00–3.13)	1.73 (0.00–3.41)
Case	R	0.12 (0.00–1.30)	0.05 (0.00–1.20)	0.70 (0.00–1.30)	0.51 (0.00–2.50)	1.71 (0.59–2.52)
Control	R	0.09 (0.00–0.57)	0.00 (0.00–0.67)	0.48 (0.11–1.33)	0.29 (0.00–0.75)	1.89 (0.55–2.78)
**Group**	**Timepoint**	**Individual play**	**Fence-directed behaviors**	**Restlessness**	**Drinking**	**Whining**
Case	A	0.20 (0.00–3.10)	1.72 (0.54–6.48)	0.00 (0.00–1.51)	0.00 (0.00–0.40)	0.00 (0.00–0.20)
Control	A	0.10 (0.00–3.64)	2.33 (0.00–9.90)	0.00 (0.00–0.10)	0.00 (0.00–0.60)	0.00 (0.00–0.33)
Case	S	0.00 (0.00–0.30)	1.67 (0.22–12.68)	0.82 (0.00–3.90)	0.00 (0.00–0.30)	0.90 (0.00–6.58)
Control	S	0.00 (0.00–2.62)	1.32 (0.10–7.36)	0.81 (0.00–2.75)	0.00 (0.00–0.58)	0.25 (0.00–19.23)
Case	R	0.22 (0.00–2.28)	3.14 (0.29–9.84)	0.00 (0.00–1.58)	0.11 (0.00–0.38)	0.17 (0.00–3.43)
Control	R	0.00 (0.00–2.57)	1.81 (0.10–8.05)	0.00 (0.00–0.61)	0.00 (0.00–0.30)	0.00 (0.00–0.57)

A: acclimatization phase, S: separation phase, R: reunion phase.

**Table 4:** Generalized linear model predicting changes in dog behavior frequency.

Predictive Factors	B	S.E.	Sig.	Exp(B)	95% Wald Confidence Interval for Exp(B)	
					Lower	Upper
**Explore**						
[TIMEPOINT = T0]	0.909	0.1196	0.001	2.483	1.964	3.139
[TIMEPOINT = T2]	0.636	0.1343	0.001	1.889	1.452	2.459
[TIMEPOINT = T1]	0a			1		
[TIMEPOINT = T2] * [Group = Case]	0.452	0.1926	0.019	1.572	1.078	2.293
[TIMEPOINT = T2] * [Group = Control]	0a			1		
**Individual play**						
[TIMEPOINT = T0]	0.595	0.2178	0.006	1.812	1.182	2.778
[TIMEPOINT = T1]	0a			1		
[Group = Case]	−1.779	0.4327	0.001	0.169	0.072	0.394
[Group = Control]	0a			1		
[TIMEPOINT = T0] * [Group = Case]	1.032	0.3644	0.005	2.806	1.374	5.731
[TIMEPOINT = T0] * [Group = Control]	0a			1		
[TIMEPOINT = T1] * [Group = Case]	−1.032	0.3644	0.005	0.356	0.174	0.728
[TIMEPOINT = T1] * [Group = Control]	0a			1		
[TIMEPOINT = T2] * [Group = Case]	1.594	0.4097	0.001	4.923	2.205	10.99
[TIMEPOINT = T2] * [Group = Control]	0a			1		
[Group = Case] * COPEPTIN pg/mL	0.001	0.0007	0.043	1.001	1	1.003
[Group = Control] * COPEPTIN pg/mL	−0.002	0.0011	0.026	0.998	0.995	1
**Spontaneous interactions with a stranger**						
[Group = Case]	0.854	0.3191	0.007	2.348	1.256	4.389
[Group = Control]	0a			1		
[Group = Case] * COPEPTIN pg/mL	−0.002	0.0008	0.005	0.998	0.996	0.999
**Spontaneous interactions with the owner**						
[Group = Case]	1.017	0.2743	0.001	2.765	1.615	4.733
[Group = Control]	0a			1		
[Group = Case] * COPEPTIN pg/mL	−0.003	0.0009	0.003	0.997	0.996	0.999
**Fence-directed behaviors**						
[TIMEPOINT=T0]	−0.387	0.0961	0.001	0.679	0.562	0.82
[TIMEPOINT=T2]	−0.241	0.1047	0.021	0.786	0.64	0.965
[TIMEPOINT=T1]	0a			1		
[TIMEPOINT = T2] * [Group = Case]	−0.397	0.1523	0.009	0.672	0.499	0.906
[TIMEPOINT = T2] * [Group = Control]	0a			1		
**Restlessness**						
[TIMEPOINT = T0]	−1.303	0.3102	0.001	0.272	0.148	0.499
[TIMEPOINT = T2]	−0.642	0.2762	0.020	0.526	0.306	0.904
[TIMEPOINT = T1]	0a			1		
[TIMEPOINT = T2] * [Group = Case]	−1.054	0.4339	0.015	0.348	0.149	0.816
[TIMEPOINT = T2] * [Group = Control]	0a			1		
**Drinking**						
[TIMEPOINT = T0]	0.905	0.3181	0.004	2.472	1.325	4.61
[TIMEPOINT = T2]	0.709	0.3636	0.051	2.031	0.996	4.142
[TIMEPOINT = T1]	0a			1		
[Group = Control] * COPEPTIN pg/mL	0.002	0.0011	0.080	1.002	1	1.004
**Whining**						
[TIMEPOINT = T0]	−1.158	0.2421	0.001	0.314	0.196	0.505
[TIMEPOINT = T2]	−1.427	0.2963	0.001	0.24	0.134	0.429
[TIMEPOINT = T1]	0a			1		
[TIMEPOINT = T2] * [Group = Case]	0.707	0.3487	0.043	2.027	1.024	4.016
[TIMEPOINT = T2] * [Group = Control]	0a			1		
[Group = Case] * COPEPTIN pg/mL	0.002	0.0006	0.004	1.002	1.001	1.003

B: regression coefficient, S.E.: standard error, Sig.: significance, Exp(B): exponentiation of the B coefficient (odds ratio), CI: confidence interval. a: set to zero because this parameter is redundant.

**Table 5:** Generalized linear model predicting changes in dog behavior duration.

Predictive Factors	B	S.E.	Sig.	Exp(B)	95% Wald Confidence Interval for Exp(B)	
					Lower	Upper
**Explore**						
[TIMEPOINT = T0]	1.095	0.2484	0.001	2.989	1.837	4.864
[TIMEPOINT = T2]	0.852	0.2809	0.002	2.344	1.352	4.066
[TIMEPOINT = T1]	0a	.	.	1	.	.
**Individual play**						
[TIMEPOINT = T0]	1.905	0.4346	0.001	6.716	2.865	15.743
[TIMEPOINT = T2]	1.932	0.431	0.001	6.904	2.966	16.067
[TIMEPOINT = T1]	0a	.	.	1	.	.
[Group = Case]	−2.186	0.9025	0.015	0.112	0.019	0.659
[Group = Control]	0a	.	.	1	.	.
[TIMEPOINT = T0] * [Group = Case]	1.633	0.632	0.01	5.121	1.484	17.673
[TIMEPOINT = T0] * [Group = Control]	0a	.	.	1	.	.
[TIMEPOINT = T2] * [Group = Case]	1.739	0.6975	0.013	5.692	1.451	22.335
[TIMEPOINT = T2] * [Group = Control]	0a	.	.	1	.	.
**Spontaneous interactions with a stranger**						
[TIMEPOINT = T0]	−0.976	0.3504	0.005	0.377	0.19	0.749
[TIMEPOINT = T2]	−1.399	0.3994	0.001	0.247	0.113	0.54
[TIMEPOINT = T1]	0a	.	.	1	.	.
[TIMEPOINT = T2] * [Group = Case]	1.404	0.5363	0.009	4.071	1.423	11.647
[TIMEPOINT = T2] * [Group = Control]	0a	.	.	1	.	.
[Group = Case] * COPEPTIN pg/mL	−0.003	0.0011	0.011	0.997	0.995	0.999
**Jumps on owner**						
[Group = Case]	1.725	0.517	0.001	5.61	2.037	15.454
[Group = Control]	0a	.	.	1	.	.
[Group = Case] * COPEPTIN pg/mL	−0.004	0.0012	0.001	0.996	0.994	0.998
**Fence-directed behaviors**						
[TIMEPOINT = T0]	−1.52	0.3342	0.001	0.219	0.114	0.421
[TIMEPOINT = T2]	−0.882	0.3568	0.013	0.414	0.206	0.833
[TIMEPOINT = T1]	0a	.	.	1	.	.

B: regression coefficient, S.E.: standard error, Sig.: significance, Exp(B): exponentiation of the B coefficient (odds ratio), CI: confidence interval, a: set to zero because this parameter is redundant.

## Data Availability

Data will be made available upon reasonable request.

## Supplementary Materials

The following supporting information can be downloaded at: www.mdpi.com/article/10.3390/ani12151974/s1, Figure S1. The environmental setting of the test. A moment during the session with one of the dogs. The owner and the two strangers (the veterinary behaviorist and the camerawoman) are visible.; Table S1 List of questions used to collect signs of anxiety reported by owners during the behavioral consultation
