# Supplementary material for: Signs of Anxiety and Salivary Copeptin Levels in Dogs Diagnosed with Separation-Related Problems in a Short Separation Test
Source: Animals (Basel). 2022 Aug 3;12(15):1974. doi: 10.3390/ani12151974 (PMC9367405; doi:10.3390/ani12151974)

Table S1 List of questions used to collect signs of anxiety reported by owners during the behavioral consultation

Signs of anxiety reported by owners during the behavioral consultation	
1	How does your dog react to noises/thunderstorms or fireworks?
2	How does your dog react when first exposed to unfamiliar situations?
3	Does your dog show specific signs of stress (agitation, restlessness, pacing, panting, whining, barking, drooling) while on walk, in urban environments, at the vet clinic, during car travels)?
4	Does your dog shake, shiver or tremble in any situation?
5	Does your dog follow you around the house?
6	How and when does your dog look for attention?
7	Does your dog try to stay in physical contact with you when you sit down?
8	Where does your dog sleep?
9	Does your dog accept to be virtually separated from you?
10	How does your dog react if you close a door and your dog stays outside?
11	How does your dog react if you close a door and your dog stays inside without you?
12	How does your dog behave when you prepare to exit (following, getting aroused/agitated, barking/whining, displaying aggressive behaviors, becoming sad)?
13	How does your dog behave when you came back home (very reactive/aroused, jumping, barking, taking a toy, "looking guilty")?
14	What do you do/say when you go out/come back?
15	Would your dog play when home alone?
16	Would your dog eat normal food when home alone?
17	Would your dog eat treats or chew bones when home alone?
18	Could you provide a video of the dog when home alone?
19	Please describe your dog's routine (feeding times, walks, play time, schedules of owner's departure, etc.)
20	Does your dog destroy objects/furniture in your presence/absence?
21	Does your dog scratch at doors, floor, windows, curtains in your presence/absence?
22	Does your dog urinate/defecate in your presence/absence?
23	Does your dog appear very active, playful and energetic?

Figure S1. The environmental setting of the test. A moment during the session with one of the dogs. The owner and the two strangers (the veterinary behaviorist and the camerawoman) are visible. Drawing by Valentina Sammartano, from Pirrone, F.; et al. Salivary Vasopressin as a Potential Non–Invasive Biomarker of Anxiety in Dogs Diagnosed with Separation–Related Problems. Animals 2019, 9, 1033. https://doi.org/10.3390/ani9121033.

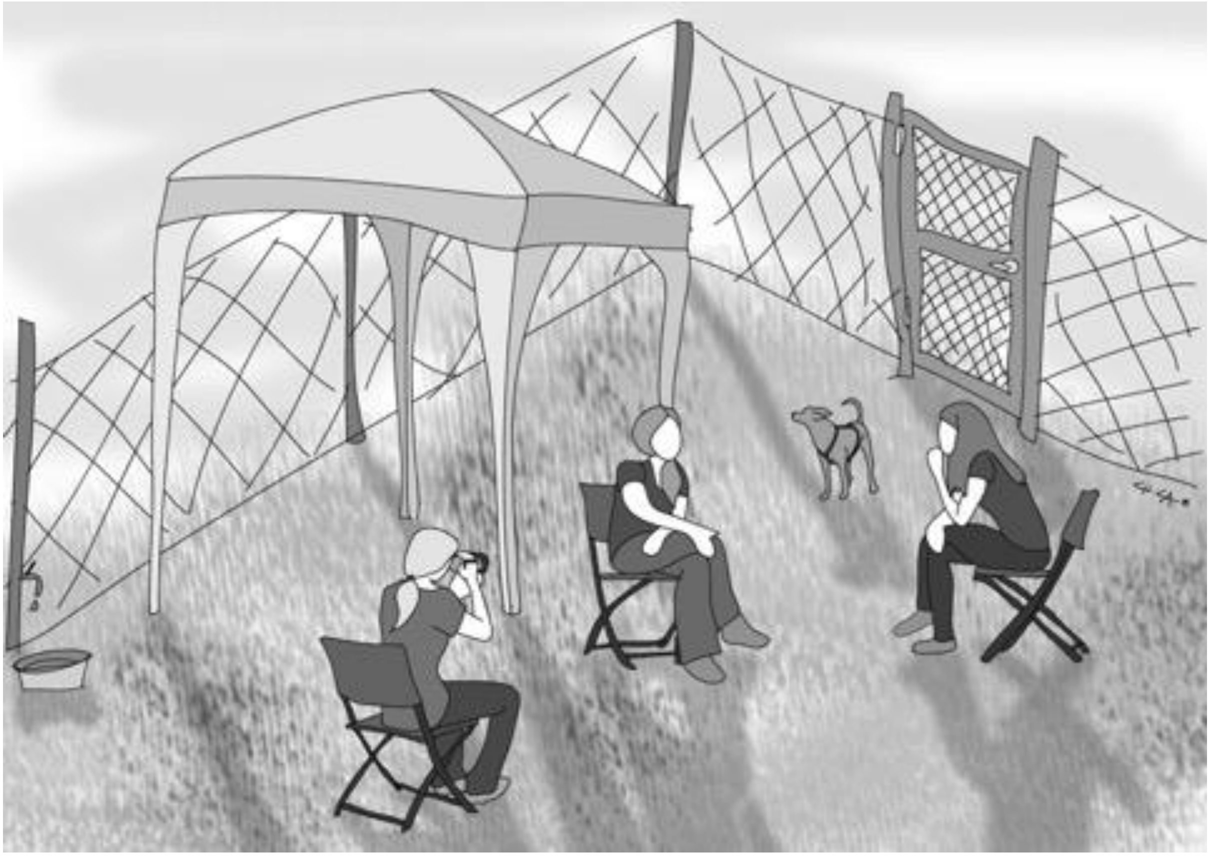

Supplement: Supplementary file 1 [file animals-12-01974-s001.zip › animals-1776440-supplementary.pdf]
